# Liao ning virus in China

**DOI:** 10.1186/1743-422X-8-282

**Published:** 2011-06-08

**Authors:** Zhi Lu, Hong Liu, Shihong Fu, Xinjun Lu, Qiang Dong, Song Zhang, Suxiang Tong, Minghua Li, Wenjuan Li, Qing Tang, Guodong Liang

**Affiliations:** 1 State Key Laboratory for Infectious Disease prevention and Control, Institute for Viral Disease Control and Prevention, Chinese Center for Disease Control and Prevention, Beijing, People's Republic of China; 2 Xinjiang Center for Disease Control and Prevention, Xinjiang, People's Republic of China

## Abstract

**Background:**

Liao ning virus is in the genus Seadornavirus within the family Reoviridae and has a genome composed of 12 segments of double-stranded RNA (dsRNA). It is transmitted by mosquitoes and only isolated in China to date and it is the only species within the genus Seadornavirus which was reported to have been propagated in mammalian cell lines. In the study, we report 41 new isolates from northern and southern Xinjiang Uygur autonomous region in China and describe the phylogenetic relationships among all 46 Chinese LNV isolates.

**Findings:**

The phylogenetic analysis indicated that all the isolates evaluated in this study can be divided into 3 different groups that appear to be related to geographic origin based on partial nucleotide sequence of the 10th segment which is predicted to encode outer coat proteins of LNV. Bayesian coalescent analysis estimated the date of the most recent common ancestor for the current Chinese LNV isolates to be 318 (with a 95% confidence interval of 30-719) and the estimated evolutionary rates is 1.993 × 10^-3 ^substitutions per site per year.

**Conclusions:**

The results indicated that LNV may be an emerging virus at a stage that evaluated rapidly and has been widely distributed in the north part of China.

## Findings

Liao ning virus (LNV) which is the member within the genus Seadornavirus family Reoviridae was composed of 12 double-stranded RNA (dsRNA). The Seadornaviruses include three species, Banna virus (BAV), Kadipiro virus (KDV) and LNV [1]. BAV was initially isolated from patients with encephalitis and appears to be pathogenic to humans [2]. Though there have been no confirmed reports of human disease due to LNV, this virus replicates in mammalian cells and causes viraemia and haemorrhage in mice [1], suggesting LNV may be pathogenic to humans or animals [1]. Recently, LNV isolates from mosquitoes have been obtained from several regions in north China [3-5]. Here, we report additional isolates from northern and southern Xinjiang province and describe the phylogenetic relationships among Chinese LNV isolates.

**Figure 1:**
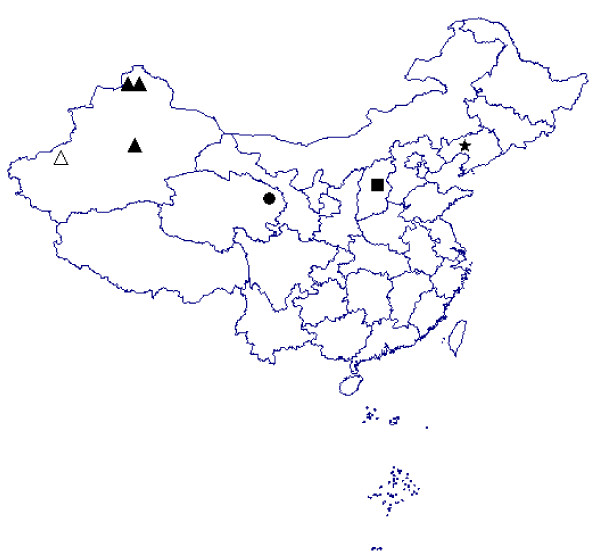
**Location of new LNVs isolated in China**. Δ Xinjiang, 2006 ▲ Xinjiang, 2007 ▲▲ Xinjiang, 2008 ● Qinghai, 2007 ■ Shanxi, 2007 ★ Liaoning, 1997

A total of 41 new LNV isolates were obtained from mosquitoes during 2006 to 2008 at sites in Xinjiang province (Figure 1). Mosquito samples were collected from July to September during 2006 to 2008 from several sites in Xinjiang province (Latitude39°to 47°N, Longitude 75° to 86°E). Mosquitoes were collected in the evening hours using UV-light traps placed in livestock sheds near human houses. Mosquitoes were sorted into pools of 50 to 100 specimens according to species. Viruses were isolated and 41 new LNV isolates were identified using previously described procedures [5]. Trizol reagent (Invitrogen, Cat No. 10296-028) was used to extract total RNA. cDNA was prepared using Ready-to-Go™ You prime First-Strand Beads Kit (Amersham Biosciences Co.) according to the manufacturer's protocol. A 576 bp gene fragment from the 10th segment was amplified from the cDNA using previously published primers [1,5]. This segment was predicted to encode the LNV's outer protein which is expected to vary noticeably from one strain to another [1]. PCR products were recovered with purification kits (Qiagen) and then were sequenced directly.

The MrModeltest version 2.3 http://www.softpedia.com/get/Science-CAD/MrModeltest.shtml software program was used to examine 24 models of nucleotide substitution to determine the model most appropriate for Bayesian coalescent analysis of the LNV dataset. The general time reversible evolutionary model incorporating a gamma distribution (GTR + G) was found according to the Akaike information criterion. Both strict and relaxed (uncorrelated exponential and lognormal) molecular clocks [6] were employed to explore and the relaxed-uncorrelated lognormal molecular clock was the best supported model, according to the estimated Bayes factor using the TRACER program. Preliminary analyses revealed that the population dynamics of LNVs supported a model of Bayesian skyline models [7]. The dataset was examined with Bayesian Markov chain Monte Carlo (MCMC) methods implemented in the BEAST package using a chain length of 100,000,000 generations with 10% removed as burn- in. All runs were checked for convergence by using the TRACER program http://tree.bio.ed.ac.uk/software/tracer/. The rate of nucleotide substitutions per site per year and the date of the time of most common ancestor (TMRCA) for these viruses were estimated. Maximum clade credibility (MCC) tree was constructed and converted to a graphics format using Figtree software version 1.2.2 http://tree.bio.ed.ac.uk/software/figtree/.

**Figure 2:**
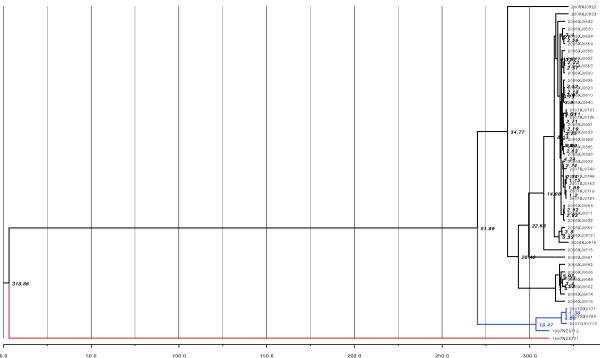
**Phylogenetic analysis of the LNVs isolated from the China**. Nucleotide of partial coding sequence of the10th segment of LNVs base maximum clade credibility (MCC) tree showing that 46 LNVs sequences from China fall into 3 groups which was colored according to different groups. The estimated TMRCA of these clades are indicated. Horizontal bar represents the scale of the estimated divergence year.

By a Bayesian MCMC approach, the mean rate of nucleotide substitution for the10th segment of Chinese LNVs was 1.993 × 10^-3 ^per site per year (95%HPD,3.664 × 10^-4 ^to 4.667 × 10^-3 ^substitutions per site per year). Based on this nucleotide substitution value, the TMRCA of Chinese LNVs is estimated to be 318 (95%HPD, 30-719) (Figure 2).

Phylogenetic analysis was conducted on 46 LNV isolates (Table 1). Of these, 41 were obtained in this study: 31 from *Culex *spp. in Kashi, southern Xinjiang in 2006; 7 from *Culex *spp. and *Aedes dorsalis *in Bayinguoleng, southern Xinjang in 2007; 3 from *Aedes dorsalis *in Aletai, northern Xinjiang in 2008(Figure 1). The other LNV isolates evaluated include: NE9712 and NE9731 isolated from *Aedes dorsalis *in Liaoning province in 1997 [1]; QH07130 isolated from *Culex modestus *in Qinghai province in 2007 [4]; SX0771 and SX0794 isolated from *Culex pipiens *and *Aedes dorsalis *in Shanxi province in 2007 [5] (Figure 1). The MCC tree shows that the LNV isolates evaluated in this study can be divided into 3 different groups that appear to be related to geographic location (Figure 2). The Xinjiang isolates are all clustered in Group A, regardless of the mosquito species from which they were isolated. Qinghai, Shanxi and one of the Liaoning isolates NE9712 are included in group B and another Liaoning isolate LNVNE9731 also from the initial isolation site of LNV was evolved independently (Figure 2). The divergence time of each lineage of the 46 LNV isolates were also estimated by Bayesian analysis. Results showed that group A (NE9731) was the oldest lineage based on the analysis of current LNVs. As well as the diverged time of Group B and Group C was 37.7 and 18.47, respectively. Data showed that the nucleotide and amino acid homology between the 41 LNV isolates are between 77.1-100% and 68.3-100%, respectively, with NE 9731 from Liaoning province the most divergent one.

**Table 1:** Information of LNV isolates in China

Province	Strain	Origin	Time of Collection	Vector
Kashi, southern Xinjiang	XJ0602, XJ0603, XJ0606, XJ0610, XJ0611, XJ0614, XJ0615, XJ0616, XJ0620, XJ0621, XJ0623, XJ0624, XJ0626, XJ0627, XJ0632, XJ0635, XJ0640, XJ0642, XJ0648, XJ0653, XJ0654, XJ0656, XJ0657, XJ0658, XJ0659, XJ0661, XJ0662, XJ0665, XJ0670, XJ0672, XJ06121	sheep house	2006.Jul-Aug	*Culex *spp.

Bayinguoleng, southern Xinjiang	XJ0719, XJ0727, XJ0735, XJ0737	piggery	2007.Jul	*Culex *spp.
	XJ0740, XJ0746, XJ0753	piggery	2007.Jul	*Aedes dorsalis*

Alertai, northern Xinjiang	XJ0815, XJ0822,	piggery	2008.Jul	*Aedes dorsalis*
	XJ0837	cow house	2008.Jul	*Aedes dorsalis*

Shanxi	SX0771	piggery	2007.Aug	*Culex pipiens pallens*
	SX0794	piggery	2007.Aug	*Aedes dorsalis*

Qinghai	QH07130	reed field	2007.Aug	*Culex modestus*

Liaoning	NE9712, NE9731	piggery	1997	*Aedes dorsalis*

Indonesia	JKT6423		1980	

Arbovirus undergoes substantially slower rates of evolution [8]. For example, the evolution rates of Dengue virus and bluetongue virus are about 10^-4^[9,10]. Our results showed that the evolution rate of LNV was 1.993 × 10^-3 ^which is faster than other arbovirus. In addition, the TMRAC of LNV was estimated to be 381 years, indicating LNV is an emerging virus and at a stage that evaluated rapidly to accommodate the host and the environment now. Also, the nucleotide and amino acid homology rate showed high identity within each group and much divergent between the groups. The geographical characteristic implies that LNV could raise natural circle in the locality though the diverged time of each group was quite recent.

In recent years, arbovirus surveillances have been done in many provinces. Japanese encephalitis virus, Tahyna virus, Getah virus and other arboviruses were isolated nationwide [11-13]. But all of the LNV isolates have been obtained only in region extending from Latitude 36°N to 47°N in the northwest to northeast part of the country. So for what reason, the virus has only been isolated from such a narrow geographic is too be studied.

LNV seemed to be at a stage of evolving rapidly than most of the arbovirus. Also it is much divergent between each subtype. Can such virus genetic differences have pathogenicity differences? LNV is pathogenic to mice, and seasonal encephalitis in the absence of Japanese encephalitis occurs in the areas where LNV is found in China [14,15], future research should investigate the potential association of LNV and human diseases.

## Competing interests

The authors declare that they have no competing interests.

## Authors' contributions

ZL, XJL, QD, SZ, SXT, MHL and WJL collected the mosquitoes sample. ZL, SHF, MHL and WJL did virus isolation, RT PCR and sequencing studies. ZL and HL did the Bioinformatics analysis. ZL, HL, QT and GDL conceived the study and drafted the manuscript. All authors read and approved the final manuscript.
